# Metabolite Screening From *Pinus pinea* Needles Reveals (+)‐Isocupressic Acid as a Key Phytotoxin for Weed Management

**DOI:** 10.1002/pca.3546

**Published:** 2025-05-30

**Authors:** Hajer Hlaili, Jesús G. Zorrilla, Maria Michela Salvatore, Mejda Abassi, Maria Teresa Russo, Miriam I. Martínez‐González, Marina DellaGreca, Alessio Cimmino, Francisco A. Macías, Anna Andolfi, Rosa M. Varela, Marco Masi

**Affiliations:** ^1^ Department of Chemical Sciences University of Naples Federico II Naples Italy; ^2^ Faculty of Science of Bizerte University of Carthage Bizerte Tunisia; ^3^ Laboratory of Forest Ecology, National Institute of Research in Rural Engineering, Water and Forests (INRGREF) University of Carthage Ariana Tunisia; ^4^ Allelopathy Group, Department of Organic Chemistry, Facultad de Ciencias, Institute of Biomolecules (INBIO) University of Cadiz Puerto Real Spain; ^5^ Department of Veterinary Medicine and Animal Production University of Naples Federico II Naples Italy

**Keywords:** allelopathy, bioprospection, by‐product valorization, GC–MS, phytochemistry, stone pine

## Abstract

**Introduction:**

Weeds are a major threat to crop productivity, competing for essential resources and often developing resistance to herbicides, which underscores the need for novel, sustainable control strategies. The valorization of agricultural and forestry underutilized byproducts, such as plant needles, presents a promising opportunity for developing eco‐friendly bioherbicides based on allelopathy.

**Objectives:**

This study investigates the phytotoxicity of *Pinus pinea* needle extracts and metabolites to evaluate their potential for controlling dicotyledonous weeds.

**Material and Methods:**

The chemical characterization of extracts and isolated compounds was performed via GC–MS, NMR, and optical methods while phytotoxicity bioassays were carried out using the herbicides Pacifica Plus (Bayer CropScience) and pendimethalin, the active ingredient in Stone Aqua (Tokyo Chemical Industry), as positive controls.

**Results:**

The dichloromethane extract exhibited the highest phytotoxicity, significantly inhibiting *Portulaca oleracea* and *Plantago lanceolata* weeds. GC–MS analysis revealed an array of aromatic compounds of interest for phytochemical research, and through bio‐guided purification, five lignans and the diterpenic acid (+)‐isocupressic acid were isolated. (+)‐Isocupressic acid showed the strongest phytotoxicity on *P. oleracea* , particularly on root growth (−83% ± 4% at 1000 μM), which could be correlated with structural moieties in its structure (fused‐ring scaffold with an exocyclic double bond, an exocyclic chain containing a double bond or hydroxyl group, and a carboxylic acid group), a number of H‐bond donors ≤ 2, and higher lipophilicity (Clog *p* = 5.11). Some lignans displayed mild inhibitory or stimulatory effects on *P. lanceolata* .

**Conclusion:**

*P. pinea* needle extracts and metabolites have demonstrated potential as natural bioherbicides for weed management. Further research is prompted to explore large‐scale applicability, environmental safety through ecotoxicological studies, and optimized formulations to enhance their practical use in sustainable agriculture.

## Graphical abstract

This study explores the phytotoxicity of *Pinus pinea* needle extracts and metabolites to evaluate their potential for controlling dicotyledonous weeds. A strategy based on GC–MS, NMR, and optical methods revealed an array of aromatic compounds which are of crucial interest for phytochemical research. Hence, a bio‐guided purification led to the isolation of five lignans and the diterpenic acid (+)‐isocupressic acid which showed a strong phytotoxicity on *Portulaca oleracea* , particularly on root growth.

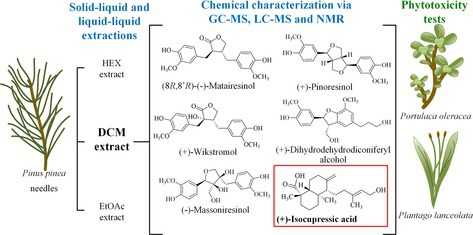

**Abbreviations**
BSTFA: *N*,*O*‐bis (trimethylsilyl)trifluoroacetamide
CC: Column chromatography
DCM: Dichloromethane
EtOAc: Ethyl acetate
HEX: *n*‐Hexane
RI: Kovats retention index
TLC: Thin‐layer chromatography
TMS: Trimethylsilyl group

## 1 Introduction

Weeds are unwanted plants that compete for resources such as nutrients, water, space, and light, leading to substantial harvest yield loss in agriculture. They are among the most persistent and harmful threats to crop productivity, highlighting the relevance of biotic stress management as the pressure to enhance crop yields and production intensifies [1, 2]. Some estimates reported that around two‐thirds of the weeds considered most relevant are broadleaved or dicotyledonous species [3], with broad leaves enabling efficient sunlight capture (outcompeting crops for resources) and deep taproot systems that ease quick recovery after physical removal [4, 5]. Although the regulation of growth processes is largely conserved between dicots and monocots, significant differences appear during their life cycle, including molecular networks and pathway interactions involved in seed development [6, 7]. Considering also that many dicotyledonous weeds have developed resistance to herbicides [8, 9], there is a clear need for the development of novel effective control agents for dicot weeds.

Synthetic herbicides have been commonly used to improve weed management, with a significant growth during the 20th century as result of the expansion of the chemical industry, which repurposed compounds originally developed for other uses, such as pigments, pharmaceuticals, or war applications [10, 11, 12]. However, immoderate consumption of such herbicides has been related to negative consequences like evolved resistance or contamination of nearby environment and waterways [13, 14]. Nowadays, sustainable farming principles aim to reduce the reliance on synthetic pesticides and herbicides, and research has focused on developing eco‐friendly herbicides designed to address and mitigate the negative impacts and limitations associated with traditional herbicides [15, 16]. A suitable point of view is provided by allelopathy, targeting phytotoxic extracts and allelochemicals as models for the development of natural herbicides [17, 18]. For example, by utilizing sustainable raw materials such as plant extracts, many have been incorporated into the production of bioherbicides [19]. In the current context, there is a growing interest in the valorization of agricultural and forestry byproducts, often treated as waste, allowing the finding of valuable resource for bioactive compounds. Pine needles are emerging as a promising material in this search [20].

Stone pine (*Pinus pinea L*.), distinctive in Mediterranean ecosystems with overall world area estimated in 600,000 ha, is a valuable species by habitat restoration programs, being the most relevant in countries like Tunisia [21, 22]. *P. pinea* is highly adaptable, tolerates calcareous soils, and is among the most resilient species in hardened superficial horizons. It holds economic and nutritional values, with forests providing protection against soil erosion due to extensive root systems and playing crucial roles in carbon sequestration, attenuating climate change impacts [23, 24, 25, 26, 27]. The well‐developed root system enhances soil quality and effectively controlles erosion in mountainous areas and contributes to the stabilization of coastal dunes. Thus, its valuable properties make this species to be widely promoted by agroforestry systems, including combinations with productive crops under intensive food production systems to address the increasing of worldwide food demand, anticipated to rise by more than 56% by 2050 [28, 29, 30, 31]. The allelopathic effect of *P. pinea* has not been well‐documented. Research on essential oils extracted through hydrodistillation and composed mainly of limonene (54.1%), α‐pinene (7.7%), (*Z*)‐caryophyllene (5.7%), β‐pinene (3.4%), and myrcene (3%) showed inhibition or delayed germination in different target weed species [32]. In addition, previous studies investigated aqueous extracts from stone pine litter on the germination and seedling performance of shrub species from the Cistaceae family [33]. Based on these findings, targeted research on the phytochemistry of *P. pinea* needles for solutions against dicotyledon weeds is encouraged to explore their valorization as bioherbicides.

In this study, several metabolites commonly found in plants have been identified from *P. pinea* needles by using a strategy which combine GC–MS, LC–MS, and NMR. Furthermore, the phytochemical properties of *P. pinea* needle extracts (collected in Tunisia) and isolated metabolites have been investigated, focusing on their effects on two dicotyledon weed species: the annual *Portulaca oleracea* and the perennial *Plantago lanceolata* . Both species could act as rapid‐growth invasive weeds on several crops worldwide, including vegetables, cereals, and pastures [34, 35, 36]. Unlike model or crop plants, which are highly selected for resilience, weed species are naturally more resistant and present a more realistic challenge for bioassays. Testing on such species ensures tested extracts and metabolites are robust enough to support further research into their practical application, bridging a critical gap in sustainable weed management.

This approach aligns with Sustainable Development Goals (SDG), particularly SDG 12: Responsible Consumption and Production, by reducing waste and utilizing sustainable raw materials, and SDG 15: Life on Land, by promoting the sustainable management of forests and promote sustainable use of terrestrial ecosystems.

## 2 Materials and Methods

### 2.1 General Experimental Procedures

^1^H NMR spectra were recorded at 400 MHz on a Bruker 400 Anova Advance (Karlsruhe, Germany) spectrometer. Samples were analyzed in deuterated chloroform (CDCl_3_) or methanol (MeOD), using solvent peaks as internal standards. Optical rotation analyses were performed using a JASCO P‐1010 polarimeter (Tokyo, Japan). Electrospray ionization mass spectra (ESI‐MS) were performed using a LC–MS TOF system AGILENT 6230B (Agilent Technologies, Milan, Italy), HPLC 1260 Infinity. Pure compounds were dissolved in methanol (MeOH) and analyzed operating in positive ionization mode. The source temperature was kept at 120°C and the desolvating gas at 250°C. A Phenomenex C_18_ reversed‐phase column Lichrocart (250 × 4.6 mm i.d.; 5 μm) was used. The elution was performed with acetonitrile: H_2_O 85:15 (formic acid 0.1%) for 12 min. HPLC flow rate was 300 μL/min. Physicochemical descriptors were calculated by ChemBioDraw Ultra 21.0 and SwissADME softwares [37, 38, 39].

Column chromatography (CC) was performed using silica gel or C_18_‐reversed phase silica gel (Kieselgel 60, 0.063–0.200 mm, and Kieselgel 100, 90 pore size, respectively, Merck, Darmstadt, Germany). Thin layer chromatography (TLC) was performed on analytical and preparative silica gel (Kieselgel 60, F_254_, 0.25 and 0.5 mm, respectively) or reverse phase (Whatman, KC18, F_254_, 0.20 mm) (Merck, Darmstadt, Germany) plates. The spots were visualized via exposure to UV light (254 nm) and/or iodine vapors and/or by spraying first with 10% H_2_SO_4_ in methanol and then with 5% phosphomolybdic acid in ethanol, followed by heating at 110°C for 10 min. Sigma‐Aldrich Co. (St. Louis, MO, USA) supplied all the reagents and the solvents.

### 2.2 Plant Material

*P. pinea* L. needles were collected from the Tabarka region, located in the northern part of Tunisia, known for its diverse and rich flora. The sampling and identification of the species were carried out under the guidance and supervision of Professor Mejda Abassi (National Institute of Research in Rural Engineering, Water and Forests‐INRGREF, Tunisia). The needles were dried and grounded finely to increase the surface area for subsequent extraction processes, ensuring optimal recovery of bioactive compounds. The processed needle powder was then stored in paper bags at standard laboratory temperature, in a dry and dark environment, until use.

### 2.3 Extraction and Isolation

Dried and minced *P. pinea* L. needles (900 g) were macerated using a hydroalcoholic mixture (1.5 L) of H_2_O/MeOH (1/1, *v/v*) at room temperature for 24 h. The hydroalcoholic suspension was centrifuged at 7000 rpm, and the supernatant was extracted with *n*‐hexane (HEX; 1 L x 3), CH_2_Cl_2_ (DCM; 1 L × 3), and after removing methanol under reduced pressure, with ethyl acetate (EtOAc; 1 L × 3) obtaining 0.4 g (HEX), 3.4 g (DCM), and 10.2 g (EtOAc) of organic extracts.

The residue of the DCM extract was purified by CC eluted with CHCl_3_/*i*‐propanol (9/1, *v/v*) yielding 11 homogeneous fractions (F1–F11) (Figure S1).

The residues of F3 (398.2 mg) and F4 (303.5 mg) were combined and further purified by CC eluted with CH_2_Cl_2_/MeOH (9.5/0.5, *v/v*) yielding nine homogeneous fractions (A.1–A.9). The residue of A.2 (111.23 mg) was further purified by two successive steps of TLC on direct and reverse phase eluted with CHCl_3_/*i*‐propanol (9/1, *v/v*) and acetonitrile/H_2_O (1/1, *v/v*) yielding three pure compounds identified as (+)‐pinoresinol (**4**, 6.98 mg), (8*R*,8′*R*)‐(−)‐matairesinol (**1**, 36.12 mg), and (+)‐wikstromol (**2**, 4.48 mg). The residue of A.3 (101.92 mg) was further purified by two successive steps of TLC on direct phase eluted with *n*‐hexane/acetone (6/4, *v/v*) and CHCl_3_/*i*‐propanol (9/1, *v/v*) yielding a pure compound identified as (+)‐isocupressic acid (**6**, 5.18 mg).

The residues of F8 (420.26 mg), F9 (197.3 mg), and F10 (300.3 mg) were combined and further purified by CC on reverse phase eluted with acetonitrile/H_2_O (1/1, *v*/*v*) yielding seven homogeneous fractions (B.1‐B.7). The residue of B.1 (38.5 mg) was further purified by TLC on direct phase eluted with CHCl_3_/*i*‐propanol (9/1, *v/v*) yielding a pure compound identified as (−)‐massoniresinol (**3**, 10.1 mg). The residue of B.2 (110.5 mg) was further purified by TLC on reverse phase eluted with acetonitrile/H_2_O (1/1, *v*/*v*) yielding a pure compound identified as (+)‐dihydrodehydrodiconiferyl alcohol (**5**, 8.8 mg).

### 2.4 Structural Characterization of Compounds

*(8R,8′R)‐(−)‐Matairesinol* (**1**): ^1^H NMR spectrum (Figure S2) was in agreement with data previously reported [40]. ESI‐MS (+) *m/z*: 739 [2 M + Na]^+^ and 381 [M + Na]^+^ (Figure S3) are consistent for a compound with the molecular formula C_20_H_22_O_6_. [α]_D_^25^–25.0 (*c* 0.5, CHCl_3_), [α]_D_^20^–24.7 lit [41]. The EI mass spectrum at 70 eV of matairesinol (**1**), 2TMS, is reported in Figure S4.

*(+)‐Wikstromol* (**2**): ^1^H NMR spectrum (Figure S5) was in agreement with data previously reported [42]. ESI‐MS (+) *m/z*: 771 [2 M + Na]^+^ (Figure S6) is consistent for a compound with the molecular formula C_20_H_22_O_7_. [α]_D_^25^ + 29.8 (*c* 0.37, CHCl_3_), [α]_D_^20^ + 29.6 lit [43]. The EI mass spectrum at 70 eV of wikstromol (**2**), 3TMS, is reported in Figure S7.

*(−)‐Massoniresinol, also known as vladinol A* (**3**): ^1^H NMR spectrum (Figure S8) was in agreement with data previously reported [44]. ESI‐MS (+) *m/z*: 807 [2 M + Na]^+^, and 415 [M + Na]^+^ (Figure S9) are consistent for a compound with the molecular formula C_20_H_24_O_8_. [α]_D_^25^–30.7 (*c* 0.79, MeOH), [α]_D_^25^–31.4 lit [45].

*(+)‐Pinoresinol* (**4**): ^1^H NMR spectrum (Figure S10) was in agreement with data previously reported [46]. ESI‐MS (+) *m/z*: 739 [2 M + Na]^+^ and 381 [M + Na]^+^ (Figure S11) are consistent for a compound with the molecular formula C_20_H_22_O_6_. [α]_D_^25^ + 49.5 (*c* 0.53, CHCl_3_), [α]_D_^23^ + 53.1 lit [47]. The EI mass spectrum at 70 eV of pinoresinol (**4**), 3TMS, is reported in Figure S12.

*(+)‐Dihydrodehydrodiconiferyl alcohol* (**5**): ^1^H NMR spectrum (Figure S13) was in agreement with data previously reported [48]. ESI‐MS (+) *m/z*: 743 [2 M + Na]^+^, and 383 [M + Na]^+^ (Figure S14) are consistent for a compound with the molecular formula C_20_H_24_O_6_. [α]_D_^25^ + 3.5 (*c* 0.13, MeOH), [α]_D_^20^ + 3.2 (*c* 0.16, MeOH), lit [49]. The EI mass spectrum at 70 eV of dihydrodehydrodiconiferyl alcohol (**5**), 3TMS, is reported in Figure S15.

*(+)‐Isocupressic acid* (**6**): ^1^H NMR spectrum (Figure S16) was in agreement with data previously reported [50]. ESI‐MS (+) *m/z*: 343 [M + Na]^+^ and 303 [M—H_2_O + H]^+^ (Figure S17) are consistent for a compound with the molecular formula C_20_H_32_O_3_. [α]_D_^25^ + 41.3 (*c* 0.27, CHCl_3_), [α]_D_^25^ + 43 lit [51]. The EI mass spectrum at 70 eV of isocupressic acid (**6**), 2TMS, is reported in Figure S18.

### 2.5 GC–MS Analyses

Gas chromatography–mass spectrometry (GC–MS) measurements were performed on crude extracts, chromatographic fractions and pure compounds after trimethylsilylation with *N*,*O*‐bis (trimethylsilyl)trifluoroacetamide (BSTFA; Fluka, Buchs, Switzerland), with exception of the HEX extract, which was analyzed without any derivatization process. GC–MS measurements were performed with an Agilent 6850 GC (Milan, Italy), equipped with an HP‐5MS capillary column (stationary phase: 5% phenyl‐methylpolysiloxane; length: 30 m; ID: 0.25 mm; film thickness: 0.25 μm), coupled to an Agilent 5973 Inert MS detector operated in the full scan mode (*m/z* 40–550) at a frequency of 3.9 Hz and with the EI ion source and quadrupole mass filter temperatures kept, respectively, at 200°C and 250°C. Helium was used as carrier gas at a flow rate of 1 mL min^−1^. The injector temperature was 250°C, and the temperature ramp raised the column temperature from 70°C to 280°C: 70°C for 1 min, 10°C min^−1^ until reaching 170°C, and 30°C min^−1^ until reaching 280°C. Then, it was held at 280°C for 5 min. The solvent delay was 4 min. Metabolites were identified by comparing their EI mass spectra at 70 eV with mass spectra collected in the NIST 20 mass spectral library (available at https://www.nist.gov/srd/nist‐standard‐reference‐database‐1a; accessed on 6 March 2025). Moreover, the identification was supported by the Kovats retention index (RI) calculated for each analyte by the Kovats equation, using the standard *n*‐alkane mixture in the range C7‐C40.

### 2.6 Bioassays

Bioassays on etiolated wheat coleoptiles ( *Triticum aestivum* L. cv. Burgos), *P. oleracea* and *P. lanceolata* were conducted in vitro, in triplicates following previously reported protocols [52, 53]. Wheat seeds were provided by Semillas Fitó S.A. (Barcelona, Spain), and weed seeds were purchased from Cantueso Natural Seeds (Cordoba, Spain). The positives control employed were the herbicide Pacifica Plus (5% amidosulfuron, 1% sodium iodosulfuron‐methyl, and 3% mesosulfuron‐methyl in WG formulation; Bayer CropScience, Leverkusen, Germany) or the active principle of the herbicide Stone Aqua, that is, pendimethalin (Tokyo Chemical Industry, Tokyo, Japan). Statistical analyses were performed using Welch's test (*n* = 3).

## 3 Results and Discussion

### 3.1 Screening and Chemical Composition of the Needle Extracts

**FIGURE 1:**
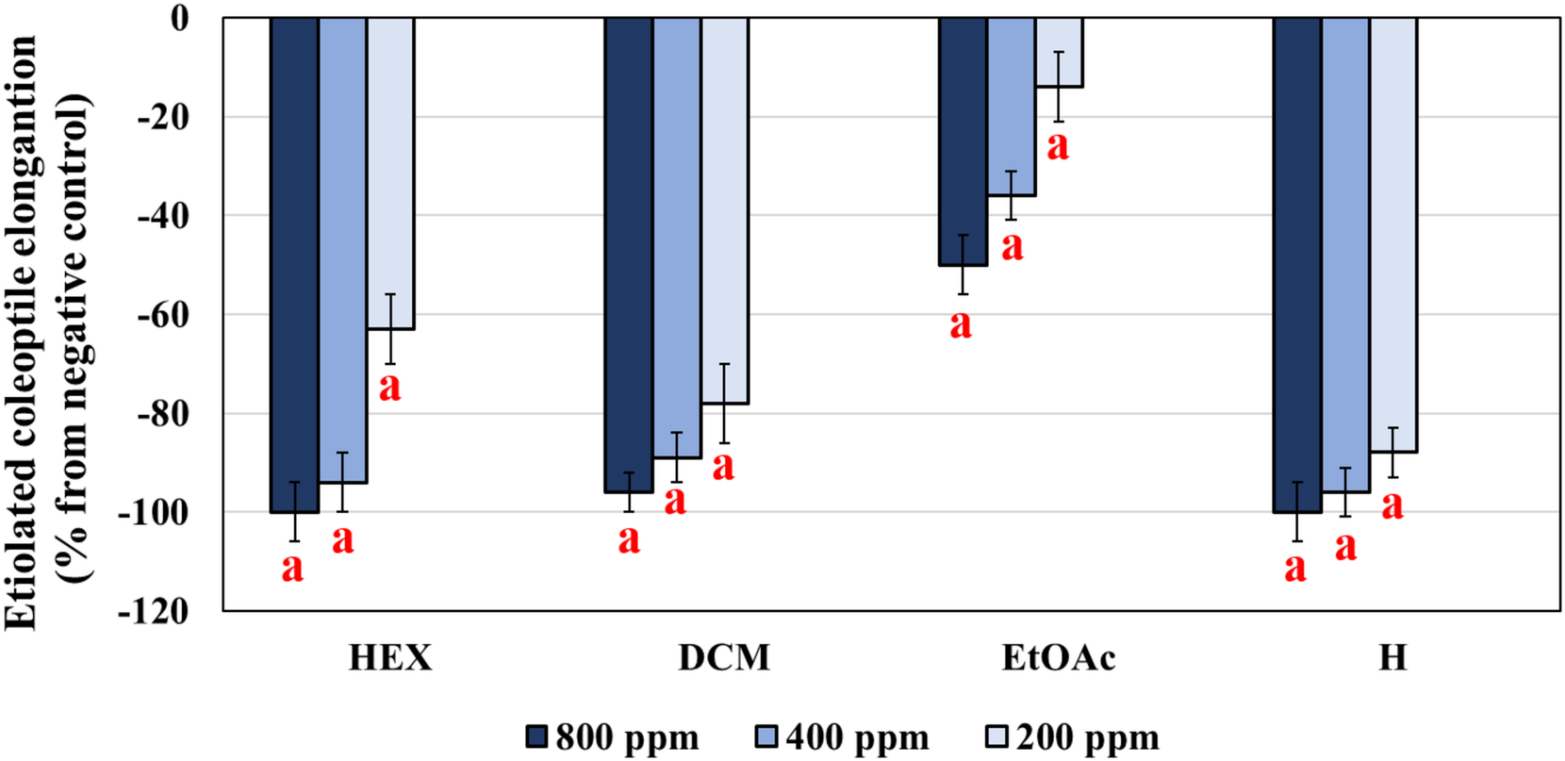
Phytotoxicity of *Pinus pinea* needle extracts obtained with *n*‐hexane (HEX), dichloromethane (DCM), and ethyl acetate (EtOAc), in the etiolated wheat ( *Triticum aestivum* L. cv. Burgos) coleoptile bioassay. Negative values indicate inhibition compared to the negative control. Error bars represent the standard error of the mean (*n* = 3). Significant differences: *p* < 0.01 (a). H: Pacifica Plus (Bayer CropScience, Leverkusen, Germany), herbicide used as positive control.

*P. pinea* needle extracts were obtained by macerating minced needles in a hydroalcoholic solution (H_2_O/methanol 1:1, *v/v*) followed by sequential extractions with solvents of increasing polarity: *n*‐hexane (HEX), dichloromethane (DCM), and ethyl acetate (EtOAc). The phytotoxicity of the HEX, DCM, and EtOAc extracts was evaluated using an etiolated coleoptile bioassay, with concentrations ranging from 800 to 200 ppm. The results, shown in Figure 1, demonstrate a clear concentration‐dependent phytotoxicity in all cases.

The HEX and DCM extracts exhibited significant phytotoxicity (Figure 1), with nearly 100% inhibition at the highest concentration (800 ppm). Both extracts retained high inhibitory effects at 400 ppm (94% ± 6% and 89% ± 5%, respectively). Notably, at 200 ppm, the DCM extract showed greater activity (78% ± 8% inhibition) compared to the HEX extract (63% ± 7% inhibition). These results highlight the superior phytotoxic potential of the DCM extract, displaying a profile similar to that of the commercial herbicide Pacifica Plus (Figure 1).

In contrast, the EtOAc extract, while exhibiting phytotoxicity, was significantly less potent than the HEX and DCM extracts, achieving only 50% ± 6% inhibition at 800 ppm (Figure 1). This marked difference in activity highlights the greater effectiveness of the less polar HEX and DCM extracts in inhibiting coleoptile elongation.

Explorative analyses via GC–MS have been carried out on the crude extracts (i.e., HEX, DCM and EtOAc extracts) to investigate their chemical composition (Figure S0). The identified metabolites and their distribution in the three examined crude extracts are reported in Table 1. The HEX extract contained a variety of metabolites, including terpenoids, aromatic compounds, and fatty acid derivatives which are commonly found in plants (Table 1). Some metabolites in the HEX extract, such as eugenol and medium‐chain fatty acids, have previously been associated with phytotoxicity, which may explain the phytotoxicity observed for this extract [54, 55]. The DCM extract was rich in aromatic compounds (Table 1), many of which have also shown phytotoxic properties [56, 57, 58]. The less active extract (EtOAc) also contained different organic acids and phenolic compounds, denoting a more complex chemical profile (Table 1) that may contribute to its lower phytotoxicity.

**TABLE 1:** Distribution of metabolites in the crude extracts from *Pinus pinea* needles identified by GC–MS analysis.

Compound	RI	*n*‐Hexane extract	Dichloromethane extract	Ethyl acetate extract
Lactic acid, 2TMS	915			+
Linalool oxide	1076	+		
Isobutyric acid, 3‐hydroxy, 2TMS	1175			+
Epoxylinalol	1182	+		
Glycerol, 3TMS	1285		+	+
Phenylacetic acid, TMS	1308		+	
Succinic acid, TMS	1320			+
Eugenol	1367	+		
Malic acid, 3TMS	1499			+
Salicylic acid, 2TMS	1530		+	
Pyroglutamic acid, 2TMS	1537			+
Dihydroactinidiolide	1558	+		
Lauric acid	1579	+		
Caryophyllene oxide	1608	+		
4‐Hydroxybenzoic acid, 2TMS	1636		+	+
Benzoic acid, 2,3‐hydroxy, 3TMS	1761			+
Vanillic acid, 2TMS	1778		+	+
Dodecanoic acid, 12‐hydroxy‐, methyl ester	1807	+		
Shikimic acid, 4TMS	1821		+	+
Protocatechuic acid, TMS	1828		+	
Pinitol, 5TMS	1864			+
Quinic acid, 5TMS	1867			+
Syringic acid, 2TMS	1910		+	
Palmitic acid, methyl ester	1928	+		
Cinnamic acid, 4 hydroxy, 2TMS (4‐coumaric acid, 2TMS)	1952		+	+
Palmitic acid	1972	+		
Gallic acid, 4TMS	1975			+
7,10‐Octadecadienoic acid, methyl ester	2100	+		
Cinnamic acid, 4‐hydroxy‐3‐methoxy, 2TMS (isoferulic acid, 2TMS)	2105		+	
Linolenic acid, methyl ester	2120	+		
Caffeic acid, 3TMS	2137			+
15‐Hydroxydehydroabietic acid, methyl ester	2596	+		
Catechine, 5TMS	2921			+
Epigallocatechin, 6TMS	2969			+
Methyl 4‐[2‐hydroxy‐2‐(4‐hydroxy‐3‐methoxyphenyl)‐1‐(hydroxymethyl)ethoxy]‐3‐methoxybenzoate, 3TMS	3001		+	+
Dihydrodehydrodiconiferyl alcohol, 3TMS	3132		+	

*Note:* “+” indicates the identification of the compound.

Abbreviations: RI, Kovats retention index; TMS, trimethylsilyl group.

### 3.2 Phytochemical Study of the Most Active Extract Against Dicotyledonous Weeds

The results obtained in the coleoptile bioassay, the amounts of extract and the chemical composition determined via GC–MS, prompted further investigations into the DCM extract, whose phytotoxicity was investigated against *P. oleracea* and *P. lanceolata* weeds.

**FIGURE 2:**
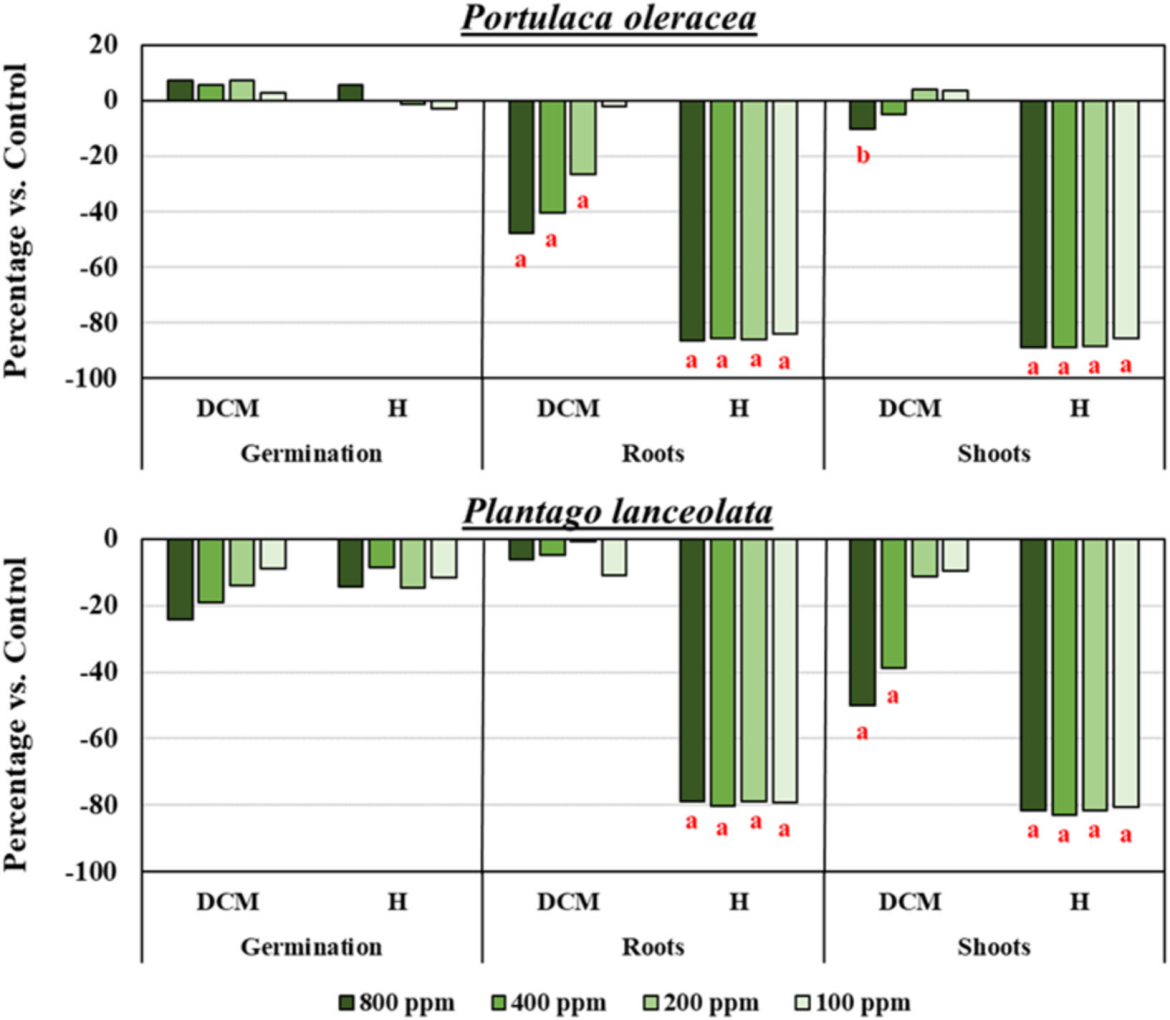
Phytotoxicity of the dichloromethane (DCM) extract of *Pinus pinea* needles against germination, root growth, and shoot growth of *Portulaca oleracea* and *Plantago lanceolata* weeds. Positive values indicate stimulation relative to the negative control, while negative values indicate inhibition. Significant differences: *p* < 0.01 (a), or 0.01 < *p* < 0.05 (b). H: pendimethalin, the active ingredient of the herbicide Stone Aqua (Tokyo Chemical Industry, Tokyo, Japan), used as positive control.

The DCM extract demonstrated potential for controlling these weeds, inhibiting root growth in *P. oleracea* at concentrations of 800–200 ppm and shoot growth in *P. lanceolata* at concentrations of 800–400 ppm (Figure 2). These observed phytotoxic effects indicate that the DCM extract contains bioactive compounds capable of targeting different plant physiological processes, particularly those involved in root and shoot development. The differing responses between both weed species may be due to variations in the absorption, translocation, or metabolism of the bioactive compounds. To further investigate and identify the specific metabolites responsible for the observed phytotoxicity, the DCM extract was subjected to bioguided purification.

**FIGURE 3:**
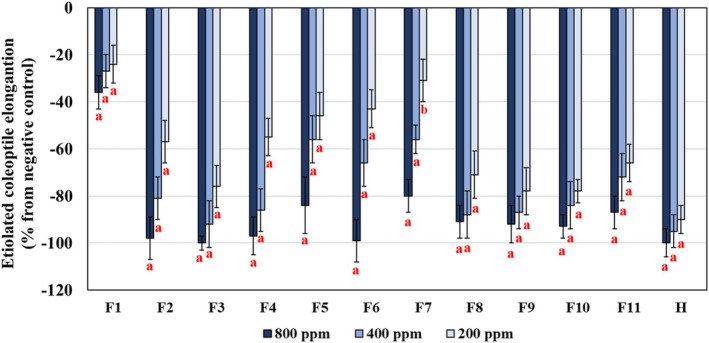
Phytotoxicity on etiolated wheat ( *Triticum aestivum* L. cv. Burgos) coleoptile bioassay of the fractions obtained from the fractionation of the dichloromethane *Pinus pinea* needle extract (F1–F11). Negative values indicate inhibition vs. the negative control. Error bars represent the standard error of the mean (*n* = 3). Significant differences: *p* < 0.01 (a), or 0.01 < *p* < 0.05 (b). H: Pacifica Plus (Bayer CropScience, Leverkusen, Germany), herbicide employed as positive control of the bioassay.

The DCM extract was fractioned on silica gel CC, yielding 11 fractions (F1‐F11) that showed different levels of phytotoxicity in the coleoptile bioassay (Figure 3). GC–MS analysis of these fractions showed the presence of several lignans (e.g., matairesinol, wikstromol, pinoresinol, isolariciresinol, and secoisolariciresinol) which are an ample class of phenylpropanoids (see Table S1). Among the metabolites identified, coniferyl alcohol, a monolignol representing the main precursor in the biosynthesis of lignans, and several cinnamic acids also involved in the phenylpropanoid pathways were detected [59, 60]. Lignans have attracted the attention of researchers for their wide variety of biological activities, such as antitumor, antimitotic, antiviral and cytotoxic, and phytochemical studies have revealed a potential phytotoxic role of these natural products [52, 59, 61, 62]. The GC–MS investigation of fractions F1‐F11 also revealed the presence of some terpenoids, fatty acids derivatives, phenolic acids, and phenolic alcohols (see Table S1), compounds known to contribute to various biological activities.

**FIGURE 4:**
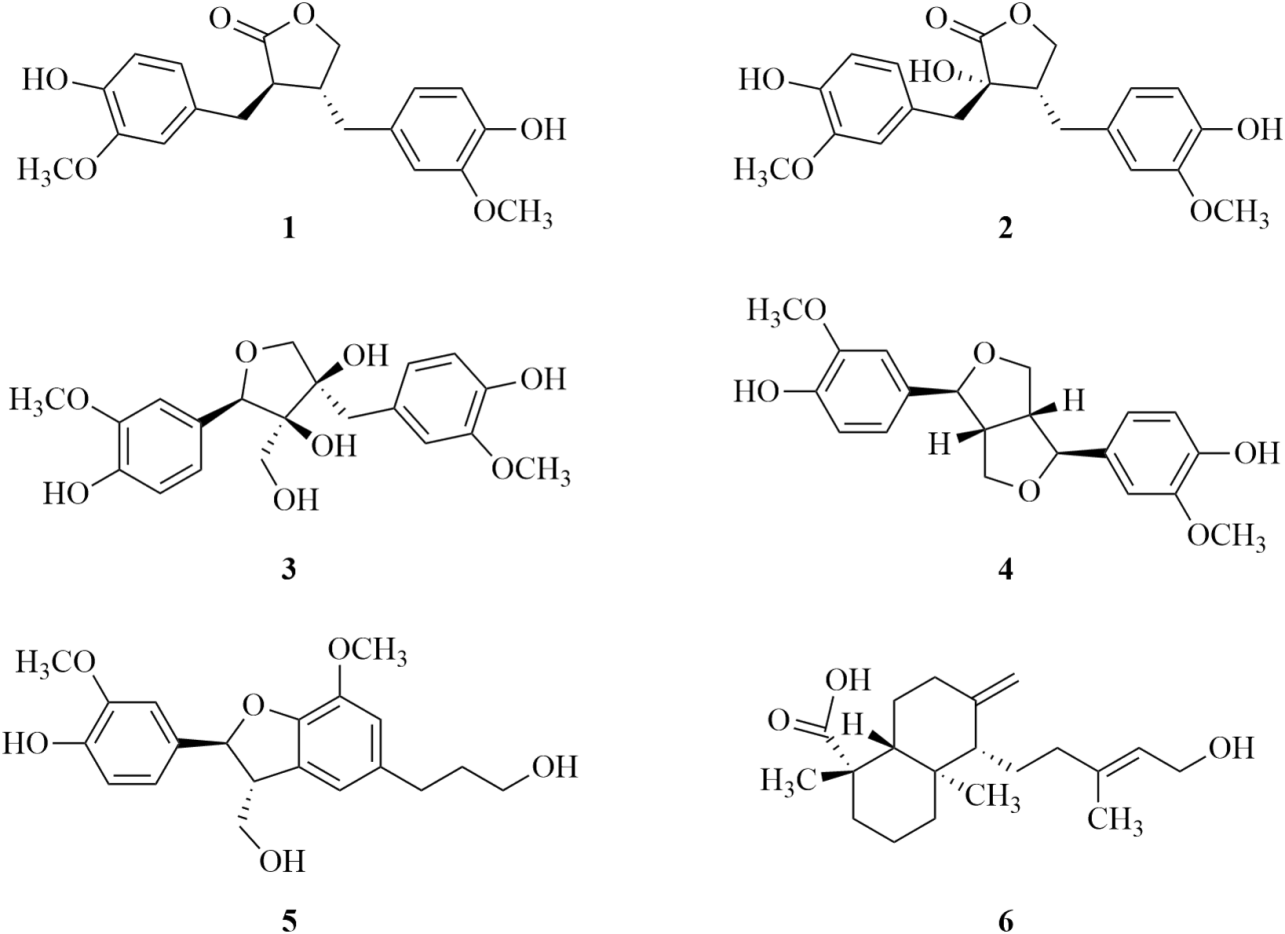
Structures of the isolated compounds from *Pinus pinea* needles: (8*R*,8′*R*)‐(−)‐Matairesinol (**1**), (+)‐wikstromol (**2**), (−)‐massoniresinol (**3**), (+)‐pinoresinol (**4**), (+)‐dihydrodehydrodiconiferyl alcohol (**5**) and (+)‐isocupressic acid (**6**).

Based on GC–MS results, yields, and activity levels, the most promising fractions were purified through different chromatographic steps by CC and TLC as described in Figure S1. Five lignans (**1–5**) and a diterpenic acid (**6**) (Figure 4) were isolated and identified as (8*R*,8′*R*)‐(−)‐matairesinol (**1**), (+)‐wikstromol (**2**), (−)‐massoniresinol (**3**), (+)‐pinoresinol (**4**), (+)‐dihydrodehydrodiconiferyl alcohol (**5**), and (+)‐isocupressic acid (**6**), comparing their spectroscopic, spectrometric, and optical data with those already reported in literature for these compounds as detailed in Section 2.4.

The isolated compounds were tested for phytotoxicity against weed species, with the exception of compound **4**, whose phytotoxicity against *P. oleracea* had been previously documented (inhibition against germination at 10^−4^–10^−6^ M) [62].

**FIGURE 5:**
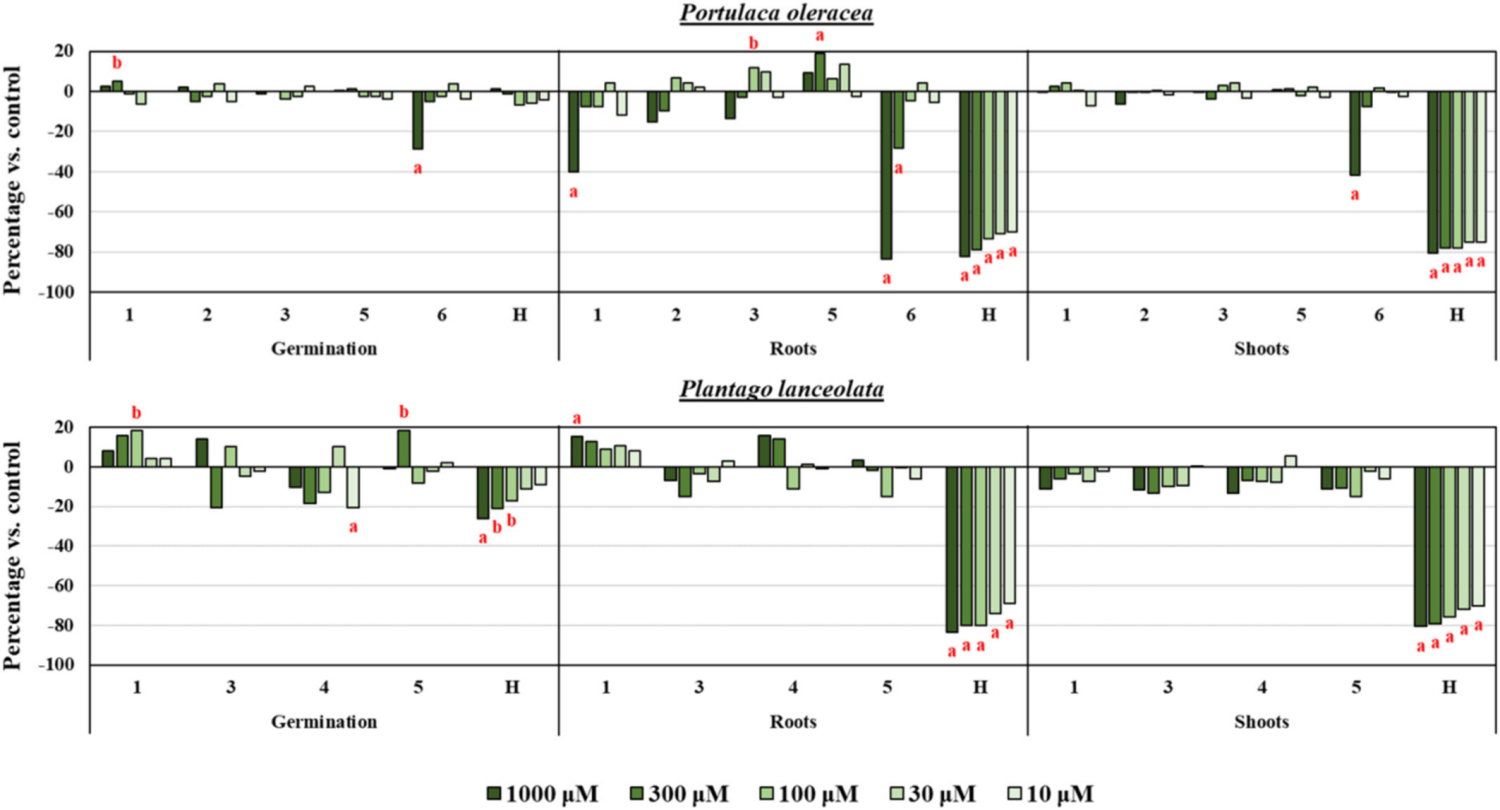
Phytotoxicity of (8*R*,8′*R*)‐(−)‐matairesinol (**1**), (+)‐wikstromol (**2**), (−)‐massoniresinol (**3**), (+)‐dihydrodehydrodiconiferyl alcohol (**5**) and (+)‐isocupressic acid (**6**) against germination, root growth, and shoot growth of *Portulaca oleracea* and phytotoxicity of **1**, **3**, (+)‐pinoresinol (**4**), and **5** against *Plantago lanceolata* . Positive values indicate stimulation relative to the negative control, while negative values indicate inhibition. Significant differences: *p* < 0.01 (a), or 0.01 < *p* < 0.05 (b). H: pendimethalin, the active ingredient of the herbicide Stone Aqua (Tokyo Chemical Industry, Tokyo, Japan), used as positive control.

In bioassay, (+)‐isocupressic acid (**6**) exhibited the most potent inhibitory effects on *P. oleracea* , especially on root growth, showing −83% ± 4% at 1000 μM, comparable to the active herbicide principle employed as positive control (Figure 5). Even at a reduced concentration of 300 μM, it maintained significant activity (−28% ± 7% root growth). At 1000 μM, compound **6** also exhibited phytotoxicity on seed germination (−29% ± 8%) and shoot growth (−42% ± 9%). These results suggest that (+)‐isocupressic acid (**6**) is a key phytotoxic metabolite in *P. pinea* needle extract, aligning with previous findings on phytotoxic metabolites active against *P. oleracea* . For instance, compounds with fused‐ring structures featuring an exocyclic double bond, such as aguerin B, and organic compounds with exocyclic chains containing a double bond or hydroxyl group, such as shogaols and gingerols from ginger roots, have demonstrated phytotoxicity against both root and shoot growth of *P. oleracea* [53, 63]. Moreover, the carboxylic acid group in (+)‐isocupressic acid (**6**) may play a crucial role in its phytotoxicity, as this functional group have been associated with the phytotoxic activity of low molecular weight metabolites, some of them involved in allelopathic plant–plant interactions [64, 65]. All these suggest that *P. pinea* needles provide a structurally advantageous metabolite of interest as model for bioherbicide development. Interestingly, GC–MS analyses of fractions F1–F11 identified a related metabolite to compound **6**, 15‐hydroxydehydroabietic acid (RI = 2623; in F3 together with compounds **1** and **4**). This compound, also a fused‐ring structure with a carboxylic acid group, could contribute to the high phytotoxic activity observed for F3 on coleoptile growth (Figure 3) and would deserve further phytochemical research.

Among the lignans (**1–5**), only (8*R*,8′*R*)‐(−)‐matairesinol (**1**) showed phytotoxicity (Figure 5), mainly root growth inhibition. A structural comparison of compound **1** with compounds **2** and **3** (Figure 4) suggests that hydroxylation of the lactone ring in this class of lignans reduced phytotoxicity against *P. oleracea* .

The efficiency of compounds as agrochemicals is also related to diverse physicochemical properties associated with mobility, stability, and bioavailability [37, 66]. Therefore, in order to discuss structure–activity relationships in relation to physicochemical properties of compounds **1–6** and the different degree of phytotoxicity observed against *P. oleracea* , it could be highlighted how the finding of phytotoxicity for compounds **1**, **4** [62], and **6** is in agreement in terms of number of H‐bond donors. Their value of two donors (Table 2) is consistent with the maximum number observed in active herbicides (≤ 2) [67], which also agrees with the higher values for the inactive compounds (**2**, **3**, and **5**). Notably, the most active compound, (+)‐isocupressic acid (**6**), has the lowest number of H‐bond acceptors (three, Table 2) and a remarked higher lipophilicity, as quantified by the Clog *p‐*values (Table 2). This last parameter could suggest that the strongest phytotoxicity of compound **6** could be related to improved solubility in cell membranes, facilitating transport to the site of action. Although compound **6** possesses a carboxylic acid group, which reduces lipophilicity (and thus Clog *p‐*value), its overall structure appears more favorable for phytotoxicity against *P. oleracea* than those of lignans **1–5**. Considering the previous reports, the potential negative influence of phenolic or acetylated aromatic rings in compounds **1–5** appears to be minimal, given that other metabolites containing this type of aromatic rings have shown phytotoxicity against *P. oleracea* [53]. At considering the fraction of sp^3^ carbons in the structures (Table 2), this fraction in compound **6** is significantly higher than those of compounds **1–5** (0.75 vs. 0.35–0.40), which may influence in the improved lipophilicity of compound **6**. No clear correlation between phytotoxicity and the number of rotatable bonds was observed (Table 2).

**TABLE 2:** Physicochemical properties and molecular descriptors of compounds **1–6**.

Parameter	1	2	3	4	5	6
Clog *P*	1.59	1.50	0.81	1.22	1.63	5.11
Fraction of C_sp3_	0.35	0.35	0.40	0.40	0.40	0.75
Number of H‐bond acceptors	6	7	8	6	6	3
Number of H‐bond donors	2	3	5	2	3	2
Number of rotatable bonds	6	6	6	4	7	5

Considering purified amounts, compounds **1** and **3–5** were also tested against *P. lanceolata* . Only (+)‐pinoresinol (**4**) showed significant phytotoxicity, with a −20% ± 3% inhibition against germination (Figure 5). As previously discussed against *P. oleracea* , this result also aligns with the trend of improved phytotoxicity in compounds that possess ≤ 2 H‐bond donors [67]. Interestingly, slight stimulation activity was observed for (8*R*,8′*R*)‐(−)‐matairesinol (**1**) on germination and root growth (Figure 5). Given that inhibitory effects were found for compound **1** against *P. oleracea* (Figure 5), as well as against germination and root elongation of the model species *Arabidopsis thaliana* in previous reports [68], this dual behavior could suggest a species‐specific effect, hinting at a more complex role for compound **1** as a plant metabolite. These observations could provide insights into its ecological function, potentially suggesting a dual role inhibitor/stimulant in plant–plant interactions under specific conditions.

Therefore, *P. pinea* needles have proven to be a promising source of extracts and bioactive compounds exhibiting dose‐dependent phytotoxicity, which is of interest for the development of weed management strategies. HEX and DCM extracts were the most effective, containing a variety of metabolites with potential herbicidal activity. The DCM extract exhibited strong phytotoxicity against the dicotyledon weeds *P. oleracea* and *P. lanceolata* , and through bioguided purification, five lignans and the diterpenic acid (+)‐isocupressic acid were purified, with the latter displaying the highest level of phytotoxicity, particularly against *P. oleracea* root growth. This compound could be a structurally advantageous metabolite as herbicide model, in relation to different structural moieties that could be correlated with phytotoxicity (fused‐ring scaffold with an exocyclic double bond, an exocyclic chain containing a double bond or hydroxyl group, and a carboxylic acid group), as well as a number of H‐bond donors ≤ 2 and higher lipophilicity.

Further research is needed to better understand the applicability and safety of *P. pinea* needle extracts and (+)‐isocupressic acid, particularly in contexts involving livestock and human health, as the latter and some of its liver‐metabolized products have been associated with effects on abortion and progesterone production [69, 70]. Studies on their environmental impact, including potential effects on nontarget organisms, as well as its stability and degradation products, considering that (+)‐isocupressic acid could undergo derivatization by microbial transformations, are also essential to assess its persistence and safety in agricultural systems [71, 72]. Approaches such as chemical modification or formulation development could help reduce the required application concentrations and mitigate potential ecological risks. Future work should also include pot‐scale experiments and testing the effects of needle extracts and metabolites on monocotyledonous weed species. These steps will contribute to the development of sustainable, plant‐based herbicide alternatives.

## Supporting information


**Figure S0.** Annotated Total Ion Chromatograms (TIC) of (A) *n*‐hexane, (B) CH_2_Cl_2_ (DCM) and (C) ethyl acetate (EtOAc) extracts of *Pinus pinea* needles. TMS: trimethylsilyl function, BSTFA: *N*,*O*‐bis (trimethylsilyl)trifluoroacetamide.
**Table S1.** Metabolites identified via GC–MS in chromatographic fractions (F1‐F11) obtained from CH_2_Cl_2_ (DCM) extract of *Pinus pinea* needle (RI: Kovats retention index; TMS: trimethylsilyl function). The presence of compounds is indicated with “+”.
**Figure S1.** Experimental design describing the essential steps of this study. Phytotoxicity tests are reported in green; chemical characterization is reported in blue.
**Figure S2.** ^1^H NMR spectrum of (8*R*,8′*R*)‐(−)‐matairesinol (**1**) recorded in CDCl_3_ at 400 MHz.
**Figure S3.** ESI‐MS spectrum of (8*R*,8′*R*)‐(−)‐matairesinol recorded in positive modality.
**Figure S4.** EI mass spectrum at 70 eV of matairesinol (**1**), 2TMS (RI = 2252). TMS = trimethylsilyl group.
**Figure S5.** ^1^H NMR spectrum of (+)‐wikstromol (**2**) recorded in CDCl_3_ at 400 MHz.
**Figure S6.** ESI‐MS spectrum of (+)‐wikstromol (**2**) recorded in positive modality.
**Figure S7.** EI mass spectrum at 70 eV of wikstromol (**2**), 3TMS (RI = 1903). TMS = trimethylsilyl group.
**Figure S8.** ^1^H NMR spectrum of (−)‐massoniresinol (**3**) recorded in CDCl_3_ at 400 MHz.
**Figure S9.** ESI‐MS spectrum of (−)‐massoniresinol (**3**) recorded in positive modality.
**Figure S10.** ^1^H NMR spectrum of (+)‐pinoresinol (**4**) recorded in CDCl_3_ at 400 MHz.
**Figure S11.** ESI‐MS spectrum of (+)‐pinoresinol (**4**) recorded in positive modality.
**Figure S12.** EI mass spectrum at 70 eV of pinoresinol (**4**), 3TMS (RI = 2879). TMS = trimethylsilyl group.
**Figure S13.** ^1^H NMR spectrum of (+)‐dihydrodehydrodiconiferyl alcohol (**5**) recorded in CD_3_OD at 400 MHz.
**Figure S14.** ESI‐MS spectrum of (+)‐dihydrodehydrodiconiferyl alcohol (**5**) recorded in positive modality.
**Figure S15.** EI mass spectrum at 70 eV of dihydrodehydrodiconiferyl alcohol (**5**), 3TMS (RI = 3132). TMS = trimethylsilyl group.
**Figure S16.** ^1^H NMR spectrum of (+)‐isocupressic acid (**6**) recorded in CDCl_3_ at 400 MHz.
**Figure S17.** ESI‐MS spectrum of (+)‐isocupressic acid (**6**) recorded in positive modality.
**Figure S18.** EI mass spectrum at 70 eV of isocupressic acid (**6**), 2TMS (RI = 2627). TMS = trimethylsilyl group.

## Data Availability

Data are contained within the article and the Supporting Information.
