# Supplementary material for: Metabolite Screening From Pinus pinea Needles Reveals (+)‐Isocupressic Acid as a Key Phytotoxin for Weed Management
Source: Phytochem Anal. 2025 May 30;36(8):2374–84. doi: 10.1002/pca.3546 (PMC12670206; doi:10.1002/pca.3546)
Supplement: Supplementary file 1 — Figure S0. Annotated Total Ion Chromatograms (TIC) of (A) n‐hexane, (B) CH2Cl2 (DCM) and (C) ethyl acetate (EtOAc) extracts of Pinus pinea needles. TMS: trimethylsilyl function, BSTFA: N,O‐bis (trimethylsilyl)trifluoroacetamide. Table S1. Metabolites identified via GC–MS in chromatographic fractions (F1‐F11) obtained from CH2Cl2 (DCM) extract of Pinus pinea needle (RI: Kovats retention index; TMS: trimethylsilyl function). The presence of compounds is indicated with “+”. Figure S1. Experimental design describing the essential steps of this study. Phytotoxicity tests are reported in green; chemical characterization is reported in blue. Figure S2. 1H NMR spectrum of (8R,8′R)‐(−)‐matairesinol (1) recorded in CDCl3 at 400 MHz. Figure S3. ESI‐MS spectrum of (8R,8′R)‐(−)‐matairesinol recorded in positive modality. Figure S4. EI mass spectrum at 70 eV of matairesinol (1), 2TMS (RI = 2252). TMS = trimethylsilyl group. Figure S5. 1H NMR spectrum of (+)‐wikstromol (2) recorded in CDCl3 at 400 MHz. Figure S6. ESI‐MS spectrum of (+)‐wikstromol (2) recorded in positive modality. Figure S7. EI mass spectrum at 70 eV of wikstromol (2), 3TMS (RI = 1903). TMS = trimethylsilyl group. Figure S8. 1H NMR spectrum of (−)‐massoniresinol (3) recorded in CDCl3 at 400 MHz. Figure S9. ESI‐MS spectrum of (−)‐massoniresinol (3) recorded in positive modality. Figure S10. 1H NMR spectrum of (+)‐pinoresinol (4) recorded in CDCl3 at 400 MHz. Figure S11. ESI‐MS spectrum of (+)‐pinoresinol (4) recorded in positive modality. Figure S12. EI mass spectrum at 70 eV of pinoresinol (4), 3TMS (RI = 2879). TMS = trimethylsilyl group. Figure S13. 1H NMR spectrum of (+)‐dihydrodehydrodiconiferyl alcohol (5) recorded in CD3OD at 400 MHz. Figure S14. ESI‐MS spectrum of (+)‐dihydrodehydrodiconiferyl alcohol (5) recorded in positive modality. Figure S15. EI mass spectrum at 70 eV of dihydrodehydrodiconiferyl alcohol (5), 3TMS (RI = 3132). TMS = trimethylsilyl group. Figure S16. 1H NMR spectrum of (+)‐isocupressic [file PCA-36-2374-s001.docx]

**Metabolite Screening from *Pinus pinea* Needles Reveals (+)-Isocupressic Acid as a Key Phytotoxin for Weed Management**

Hajer Hlaili ^1,2,3^, Jesús G. Zorrilla ^1,4*^, Maria Michela Salvatore ^5^, Mejda Abassi ^3^, Maria Teresa Russo ^1^, Miriam I. Martínez-González ^1,4^, Marina DellaGreca ^1^, Alessio Cimmino ^1^, Francisco A. Macías ^4^, Anna Andolfi ^1^, Rosa M. Varela ^4^ and Marco Masi ^1*^

^1^ Department of Chemical Sciences, University of Naples Federico II, 80126 Naples, Italy.

^2^ Faculty of Science of Bizerte, University of Carthage, Zarzouna, Bizerte, 7021, Tunisia.

^3^ Laboratory of Forest Ecology, National Institute of Research in Rural Engineering, Water and Forests (INRGREF), University of Carthage, Hédi Elkarray Street, Elmenzah IV, Ariana 2080, Tunisia.

^4^ Allelopathy Group, Department of Organic Chemistry, Facultad de Ciencias, Institute of Biomolecules (INBIO), University of Cadiz, 11510 Puerto Real, Spain.

^5^ Department of Veterinary Medicine and Animal Production, University of Naples Federico II, 80137 Naples, Italy.

*Correspondence: Jesús G. Zorrilla, [jesus.zorrilla@uca.es](mailto:jesus.zorrilla@uca.es); Marco Masi, [marco.masi@unina.it](mailto:marco.masi@unina.it)

**Supporting Information List**

**Figure S0.** Annotated Total Ion Chromatograms (TIC) of (**A**) *n*-hexane, (**B**) CH_2_Cl_2_ (DCM) and (**C**) ethyl acetate (EtOAc) extracts of *Pinus pinea* needles. TMS: trimethylsilyl function, BSTFA: *N*,*O*-bis(trimethylsilyl)trifluoroacetamide.

**Table S1.** Metabolites identified via GC-MS in chromatographic fractions (F1-F11) obtained from CH_2_Cl_2_ (DCM) extract of *Pinus pinea* needle (RI: Kovats retention index; TMS: trimethylsilyl function). The presence of compounds is indicated with “+”.

**Figure S1.** Experimental design describing the essential steps of this study. Phytotoxicity tests are reported in green; chemical characterization is reported in blue.

**Figure S2.** ^1^H NMR spectrum of (8*R*,8’*R*)-(-)-matairesinol (**1**) recorded in CDCl_3_ at 400 MHz.

**Figure S3.** ESI-MS spectrum of (8*R*,8’*R*)-(-)-matairesinol recorded in positive modality.

**Figure S4.** EI mass spectrum at 70 eV of matairesinol (**1**), 2TMS (RI = 2252). TMS = trimethylsilyl group.

**Figure S5.** ^1^H NMR spectrum of (+)-wikstromol (**2**) recorded in CDCl_3_ at 400 MHz.

**Figure S6.** ESI-MS spectrum of (+)-wikstromol (**2**) recorded in positive modality.

**Figure S7.** EI mass spectrum at 70 eV of wikstromol (**2**), 3TMS (RI = 1903). TMS = trimethylsilyl group.

**Figure S8.** ^1^H NMR spectrum of (-)-massoniresinol (**3**) recorded in CDCl_3_ at 400 MHz.

**Figure S9.** ESI-MS spectrum of (-)-massoniresinol (**3**) recorded in positive modality.

**Figure S10.** ^1^H NMR spectrum of (+)-pinoresinol (**4**) recorded in CDCl_3_ at 400 MHz.

**Figure S11.** ESI-MS spectrum of (+)-pinoresinol (**4**) recorded in positive modality.

**Figure S12.** EI mass spectrum at 70 eV of pinoresinol (**4**), 3TMS (RI = 2879). TMS = trimethylsilyl group.

**Figure S13.** ^1^H NMR spectrum of (+)-dihydrodehydrodiconiferyl alcohol (**5**) recorded in CD_3_OD at 400 MHz.

**Figure S14.** ESI-MS spectrum of (+)-dihydrodehydrodiconiferyl alcohol (**5**) recorded in positive modality.

**Figure S15.** EI mass spectrum at 70 eV of dihydrodehydrodiconiferyl alcohol (**5**), 3TMS (RI = 3132). TMS = trimethylsilyl group.

**Figure S16.** ^1^H NMR spectrum of (+)-isocupressic acid (**6**) recorded in CDCl_3_ at 400 MHz.

**Figure S17.** ESI-MS spectrum of (+)-isocupressic acid (**6**) recorded in positive modality.

**Figure S18.** EI mass spectrum at 70 eV of isocupressic acid (**6**), 2TMS (RI = 2627). TMS = trimethylsilyl group.


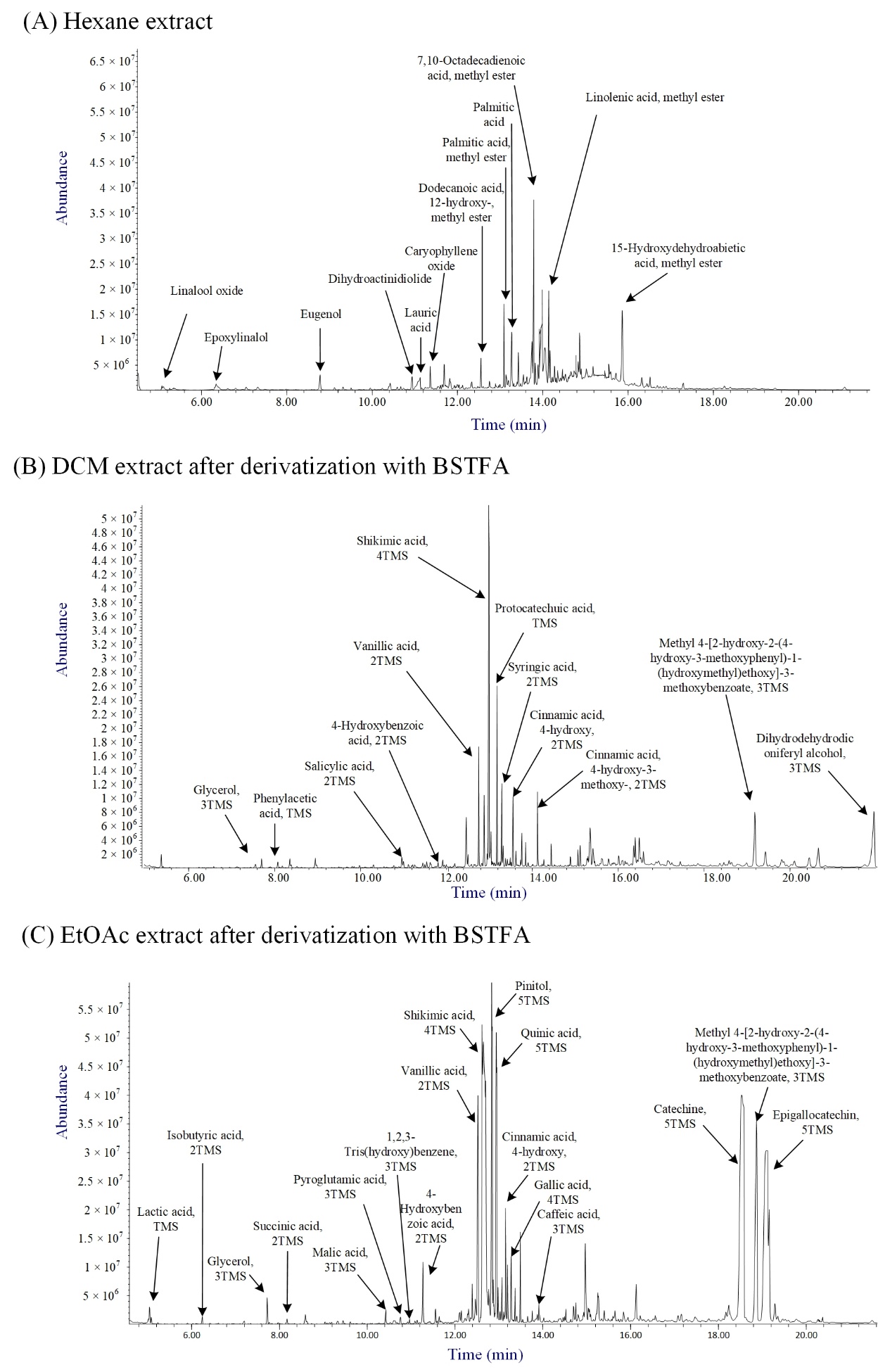


**Figure S0.** Annotated Total Ion Chromatograms (TIC) of (**A**) *n*-hexane, (**B**) CH_2_Cl_2_ (DCM) and (**C**) ethyl acetate (EtOAc) extracts of *Pinus pinea* needles. TMS: trimethylsilyl function, BSTFA: *N*,*O*-bis(trimethylsilyl)trifluoroacetamide.

**Table S1.** Metabolites identified via GC-MS in chromatographic fractions (F1-F11) obtained from the CH_2_Cl_2_ (DCM) extract of *Pinus pinea* needles (RI: Kovats retention index; TMS: trimethylsilyl function). The presence of compounds is indicated with “+”.

| **Compound** | **Chemical structure** | **RI** | **F1** | **F2** | **F3** | **F4** | **F5** | **F6** | **F7** | **F8** | **F9** | **F10** | **F11** |
| --- | --- | --- | --- | --- | --- | --- | --- | --- | --- | --- | --- | --- | --- |
| Benzoic acid, TMS |  | 1249 |  |  |  | + | + | + |  |  |  |  |  |
| Phenylacetic acid, TMS |  | 1308 |  |  |  |  | + | + | + | + |  |  |  |
| Vanillin, TMS |  | 1549 |  | + | + |  |  |  |  |  |  |  |  |
| Cinnamic acid, TMS |  | 1554 |  |  |  | + | + |  |  |  |  |  |  |
| Perillyl alcohol, TMS |  | 1576 |  |  |  |  |  |  | + |  |  |  |  |
| 4-Hydroxybenzoic acid, 2TMS |  | 1636 |  |  |  |  |  |  |  | + | + |  |  |
| Homovanillyl alcohol, 2TMS |  | 1693 |  |  |  | + |  |  |  |  |  |  |  |
| Rhododendrol, 2TMS |  | 1724 |  |  |  |  | + |  |  |  |  |  |  |
| Vanillic acid, 2TMS |  | 1778 |  |  |  |  | + | + | + | + |  |  |  |
| Loliolide, TMS |  | 1818 |  |  |  | + | + |  |  |  |  |  |  |
| Shikimic acid, 4TMS |  | 1821 | + |  |  |  |  |  |  |  |  | + | + |
| 3-Vanilpropanol, 2TMS |  | 1831 |  |  |  | + | + |  |  |  |  |  |  |
| Pinitol, 5TMS |  | 1864 |  |  |  |  |  |  |  |  |  |  | + |
| Wikstromol, 3TMS |  | 1903 |  |  |  | + |  |  |  |  |  |  |  |
| Dihydroferulic acid, 2TMS |  | 1907 |  |  |  |  |  | + |  |  |  |  |  |
| Syringic acid, 2TMS |  | 1909 |  |  |  |  |  |  | + |  |  |  |  |
| Palmitic acid, methyl ester |  | 1925 | + |  |  |  |  |  |  |  |  |  |  |
| Coniferyl alchool, 2TMS |  | 1940 |  |  |  | + |  |  |  |  |  |  |  |
| Cinnamic acid, 4-hydroxy, 2TMS (4-Coumaric acid, 2TMS) |  | 1952 |  |  |  |  | + |  |  | + | + | + |  |
| 1,11-Undecanedioic acid, 2TMS |  | 2003 |  |  |  |  |  | + |  | + |  |  |  |
| Cinnamic acid, 3-hydroxy-4-methoxy, 2TMS |  | 2107 |  |  |  |  |  | + |  | + |  | + |  |
| Isoferulic acid, 2TMS |  | 2107 |  |  |  |  |  |  | + |  | + |  |  |
| Linoleic acid methyl ester |  | 2120 | + |  |  |  |  |  |  |  |  |  |  |
| Lariciresinol, 3TMS |  | 2133 |  |  |  |  |  | + |  |  |  |  |  |
| 14-Hydroxymyristic acid, 2TMS |  | 2197 |  |  |  |  | + | + | + |  | + |  |  |
| Matairesinol, 2TMS |  | 2252 |  |  | + | + |  |  |  |  |  |  |  |
| Lanopalmitic acid, 2TMS |  | 2390 |  |  |  |  | + | + |  |  |  |  |  |
| 7-Hydroxy-8,11,13-abietatrien-19-oic acid, 2TMS |  | 2536 |  |  |  |  | + | + | + |  |  |  |  |
| 15-Hydroxydehydroabietic acid, TMS |  | 2620 |  |  | + | + |  |  |  |  |  |  |  |
| Isocupressic acid, 2TMS |  | 2627 |  |  | + |  |  |  |  |  |  |  |  |
| Pinoresinol, 2TMS |  | 2879 |  |  | + | + |  |  |  |  |  |  |  |
| Farrerol, 3TMS |  | 2929 |  | + |  |  |  |  |  |  |  |  |  |
| Isolariciresinol, 4TMS |  | 2997 |  |  |  |  |  |  |  | + |  |  |  |
| Methyl 4-[2-hydroxy-2-(4-hydroxy-3-methoxyphenyl)-1-(hydroxymethyl)ethoxy]-3-methoxybenzoate, 3TMS |  | 3001 |  |  |  |  |  |  |  |  | + | + |  |
| Secoisolariciresinol, 4TMS |  | 3045 |  |  |  |  |  |  | + |  |  |  |  |
| Peltatin, 2TMS |  | 3056 |  |  |  |  |  |  |  |  | + | + |  |
| Nonacosan-10-ol, TMS |  | 3070 | + |  |  |  |  |  |  |  |  |  |  |
| Dihydrodehydrodiconiferyl alcohol, 3TMS |  | 3132 |  |  |  |  |  |  | + | + |  |  |  |


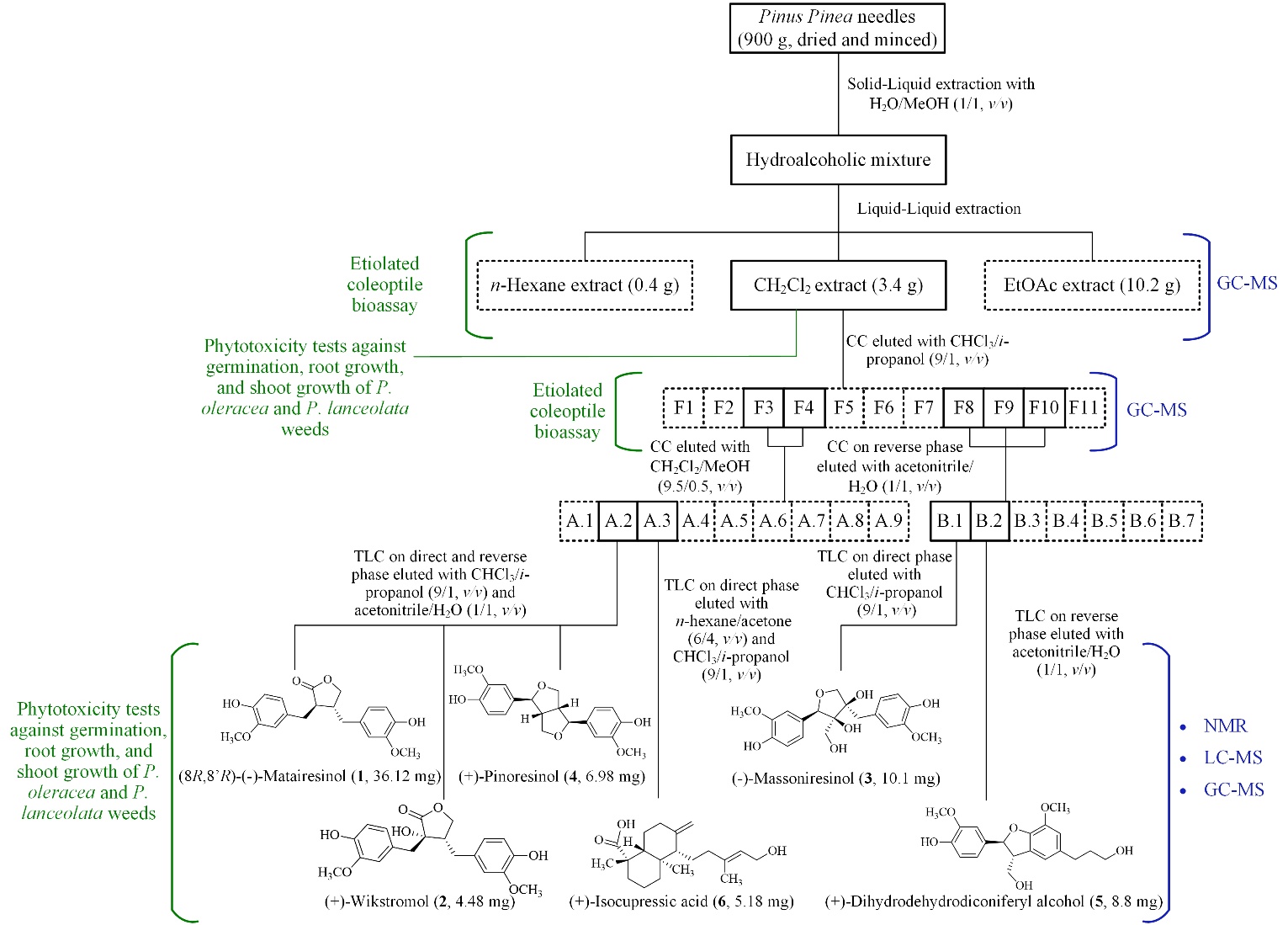


**Figure S1.** Experimental design describing the essential steps of this study. Phytotoxicity tests are reported in green; chemical characterization is reported in blue.

|  | **^1^H NMR (400 MHz, CDCl_3_, δ, ppm):** 6.81 (*d*, J = 8.2 Hz, 1H, H-5’), 6.79 (*d*, J = 8.2 Hz, 1H, H-5), 6.60 (*d*, J = 1.8 Hz, 1H, H-2’), 6.59 (overlapped *dd*, J = 1.8 Hz, 1H, H-6’), 6.50 (*dd*, J = 8.0 and 1.8 Hz, 1H, H-6), 6.40 (*d*, J = 1.8 Hz, 1H, H-2), 5.53 (*s*, 1H, -OH), 5.51 (*s*, 1H, -OH), 4.15 (*dd*, J = 9.1 and 7.4 Hz, 1H, H-9a), 3.89 (*dd*, J = 9.1 and 7.3 Hz, 1H, H-9b), 3.81 (*s*, 3H, -OCH_3_’), 3.80 (*s*, 3H, -OCH_3_), 2.94 (*dd*, J = 14.1 and 5.1 Hz, 1H, H-7’b), 2.87 (*dd*, J = 14.1 and 7.0 Hz, 1H, H-7’a), 2.63-2.44 (*m*, 4H, H_2_-7, H-8 and H-8’). |
| --- | --- |

**Figure S2.** ^1^H NMR spectrum of (8*R*,8’*R*)-(-)-matairesinol (**1**) recorded in CDCl_3_ at 400 MHz and spectroscopy data.


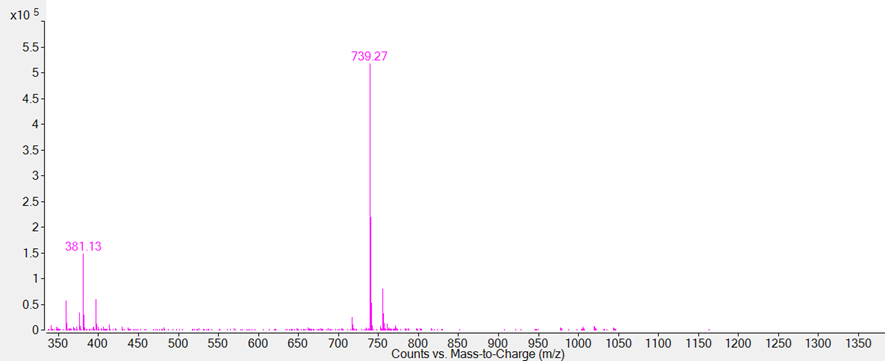


**Figure S3.** ESI-MS spectrum of (8*R*,8’*R*)-(-)-matairesinol recorded in positive modality.

**Figure S4.** EI mass spectrum at 70 eV of matairesinol (**1**), 2TMS (RI = 2252). TMS = trimethylsilyl group.

|  | **^1^H NMR (400 MHz, CDCl_3_, δ, ppm):** 6.84 (*d*, J = 8.0 Hz, 2H, H-5 and H-5’), 6.70 (*s*, 1H, H-2’), 6.63 (*d*, J = 8.0 Hz, 1H, H-6 and H-6’), 6.60 (*s*, 1H, H-2), 5.55 (*s*, 1H, -OH), 5.50 (*s*, 1H, -OH), 4.02 (*m*, 2H, H_2_-9), 3.87 (*s*, 3H, -OCH_3_), 3.85 (*s*, 3H, -OCH_3_’), 3.08 (*d*, J = 13.4, 1H, H-7’a), 2.93 (*m*, 2H, H-7a and H-7’b), 2.50 (*m*, 2H, H-7b and H-8). |
| --- | --- |

**Figure S5.** ^1^H NMR spectrum of (+)-wikstromol (**2**) recorded in CDCl_3_ at 400 MHz and spectroscopy data.


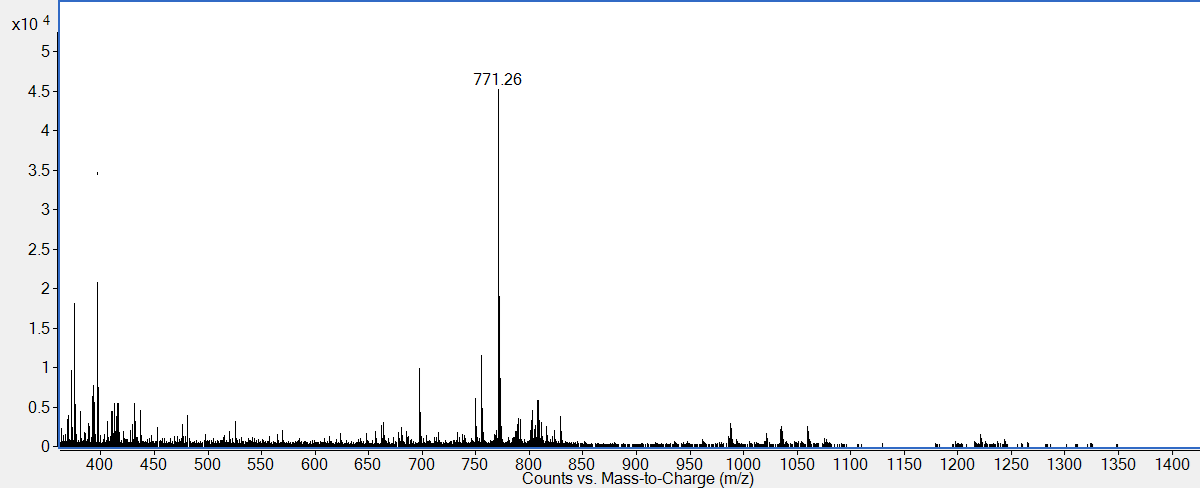


**Figure S6.** ESI-MS spectrum of (+)-wikstromol (**2**) recorded in positive modality.

**Figure S7.** EI mass spectrum at 70 eV of wikstromol (**2**), 3TMS (RI = 1903). TMS = trimethylsilyl group.

|  | **^1^H NMR (400 MHz, CDCl_3_, δ, ppm):** 6.97 (*d*, J = 1.7 Hz, 1H, H-2’), 6.90 (*d*, J = 13.0 Hz, 1H, H-5’), 6.88 (*d*, J =13.0 Hz, 1H, H-5), 6.83 (*d*, J = 1.8 Hz, 1H, H-2), 6.82 (*dd*, J = 8.0 and 1.8 Hz, 1H, H-6’), 6.77 (*dd*, J = 8.0 and 1.8 Hz, 1H, H-6), 5.65 (*s*, 1H, -OH), 5.56 (*s*, 1H, -OH), 4.98 (*s*, 1H, H-7’), 3.95 (*d*, J = 9.3 Hz, 1H, H-9a), 3.94 (*d*, J = 11.5, 1H, H-9’a), 3.89 (*s*, 6H, -OCH_3_), 3.78 (*m*, 2H, H-9b and H-9’b), 2.99 (*d*, J = 13.9 Hz, 1H, H-7a), 2.83 (*d*, J = 13.9 Hz, 1H, H-7b), 2.69 (br *s*, 2H, -OH). |
| --- | --- |

**Figure S8.** ^1^H NMR spectrum of (-)-massoniresinol (**3**) recorded in CDCl_3_ at 400 MHz and spectroscopic data.


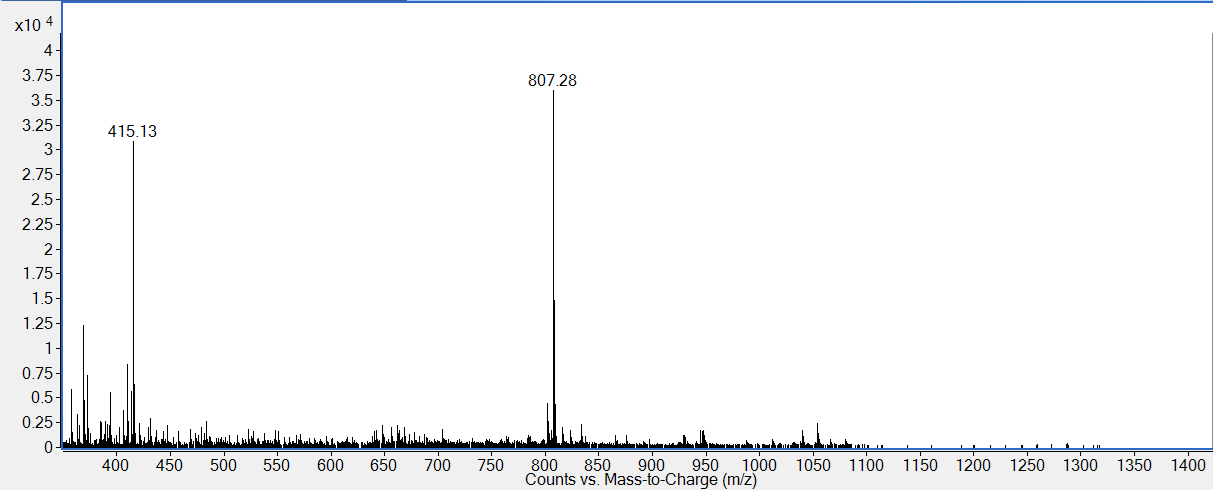


**Figure S9.** ESI-MS spectrum of (-)-massoniresinol (**3**) recorded in positive modality.

|  | **^1^H NMR (400 MHz, CDCl_3_, δ, ppm):** 6.91 (*m*, 4H, H-2 and H-5), 6.84 (*dd*, J = 8.1 and 1.4 Hz, 2H, H-6), 5.62 (2H, *s*, -OH), 4.76 (*d*, J = 4.1 Hz, 2H, H_α_), 4.27 (*dd*, J = 9.1 and 6.8 Hz, 2H, H_γ_a), 3.93 (*s*, 6H, -OCH_3_), 3.90 (*dd*, J = 9.1 and 3.5 Hz, 2H, H_γ_b), 3.10 (*m*, 2H, H_β_). |
| --- | --- |

**Figure S10.** ^1^H NMR spectrum of (+)-pinoresinol (**4**) recorded in CDCl_3_ at 400 MHz and spectroscopic data.


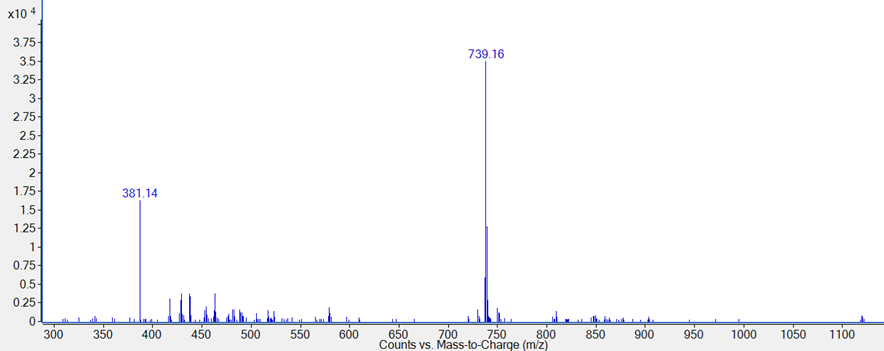


**Figure S11.** ESI-MS spectrum of (+)-pinoresinol (**4**) recorded in positive modality.

**Figure S12.** EI mass spectrum at 70 eV of pinoresinol (**4**), 3TMS (RI = 2879). TMS = trimethylsilyl group.

|  | **^1^H NMR (400 MHz, CD_3_OD, δ, ppm):** 6.93 (br *s*, 1H, H-2), 6.80 (br *d*, J = 8.1 Hz, H-6), 6.74 (*d*, J = 8.1 Hz, H-5), 6.71 (*s*, 2H, H-2’ and H-6’), 5.47 (*d*, J = 6.2 Hz, 1H, H-7), 3.83 (*s*, 3H, -OCH_3_’), 3.80 (*s*, 3H, -OCH_3_), 3.75 (*m*, 2H, H-9a and H-8), 3.55 (*t*, J = 6.4 Hz, 2H, H_2_-9’), 3.45 (*dd*, J = 12.2 and 6.2 Hz, 1H, H-9b), 2.61 (*t*, J = 7.4 Hz, 2H, H_2_-7’), 1.80 (*m*, 2H, H_2_-8’). |
| --- | --- |

**Figure S13.** ^1^H NMR spectrum of (+)-dihydrodehydrodiconiferyl alcohol (**5**) recorded in CD_3_OD at 400 MHz and spectroscopic data.


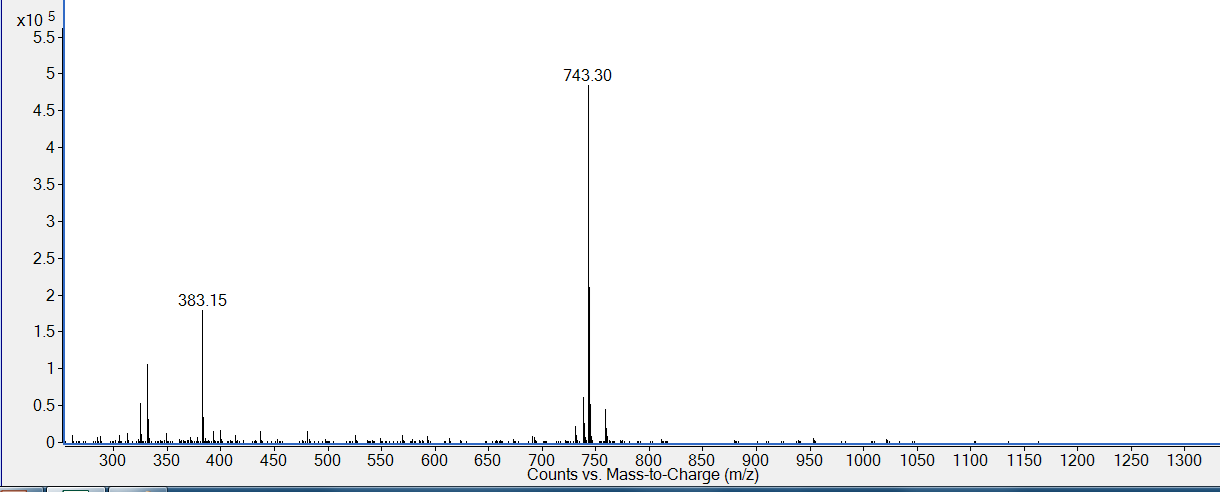


**Figure S14.** ESI-MS spectrum of (+)-dihydrodehydrodiconiferyl alcohol (**5**) recorded in positive modality.

**Figure S15.** EI mass spectrum at 70 eV of dihydrodehydrodiconiferyl alcohol (**5**), 3TMS (RI = 3132). TMS = trimethylsilyl group.


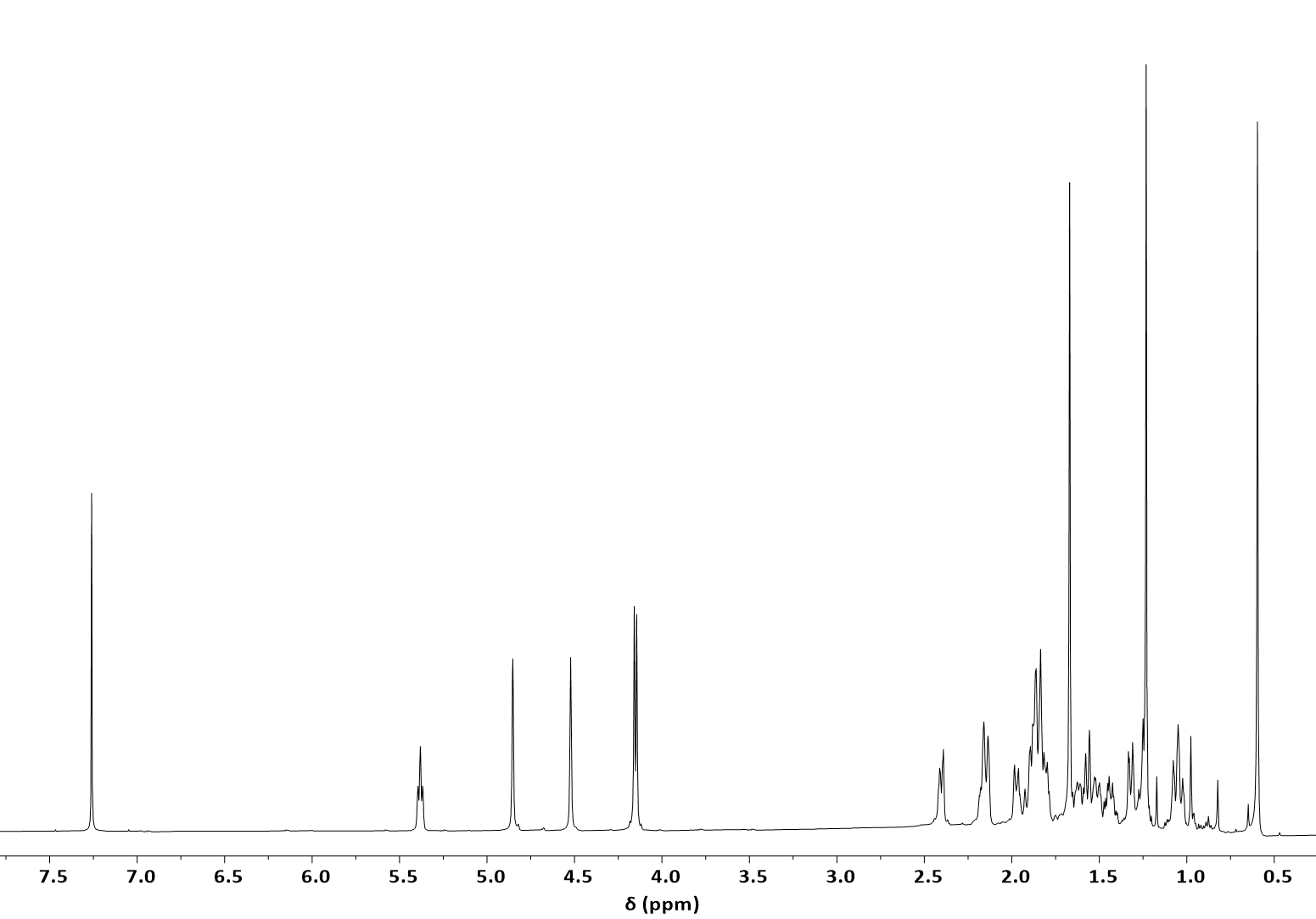


|  | **^1^H NMR (400 MHz, CDCl_3_, δ, ppm):** 5.38 (*t*, J = 6.9 Hz, 1H, H-14), 4.85 (*s*, 1H, H-17a), 4.52 (*s*, 1H, H-17b), 4.15 (*d*, J = 6.9 Hz, 2H, H_2_-15), 2.40 (*br d*, J = 8.7 Hz, 1H, H-12a), 2.15 (*m*, 2H, H-3a and H-7a), 1.98-1.79 (*m*, 6H, H-1a, H-2a, H_2_-6, H-7b and H-12b), 1.67 (*s*, 3H, H_3_-16), 1.65-1.40 (*m*, 4H, H-2b, H-9 and H_2_-11), 1.31 (*dd*, J = 11.9 and 2.7 Hz, 1H, H-5), 1.23 (*s*, 3H, H_3_-18), 1.05 (*m*, 2H, H-1b and H-3b), 0.60 (*s*, 3H, H_3_-20). |
| --- | --- |

**Figure S16.** ^1^H NMR spectrum of (+)-isocupressic acid (**6**) recorded in CDCl_3_ at 400 MHz and spectroscopic data.


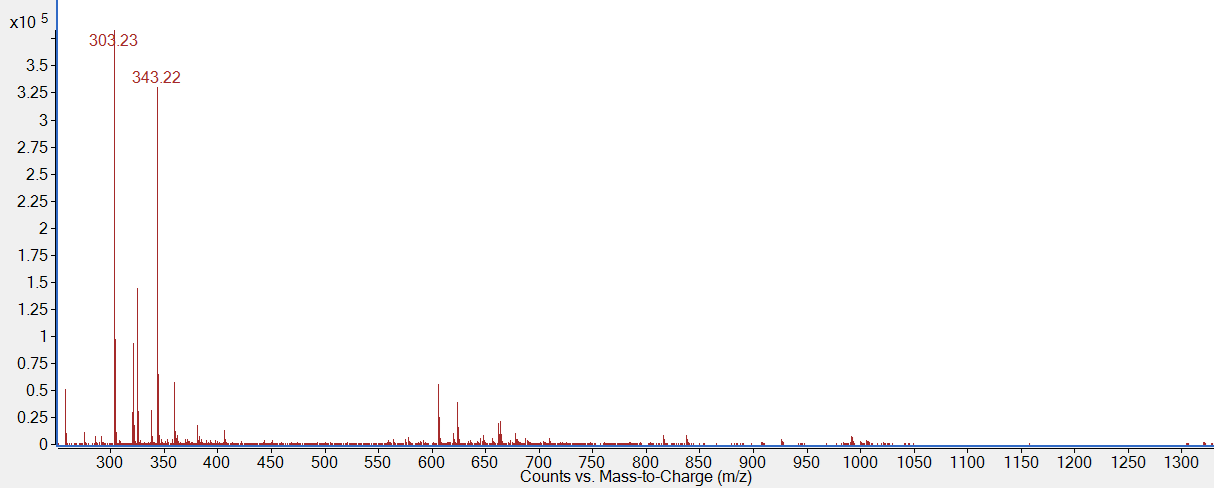


**Figure S17.** ESI-MS spectrum of (+)-isocupressic acid (**6**) recorded in positive modality.

**Figure S18.** EI mass spectrum at 70 eV of isocupressic acid (**6**), 2TMS (RI = 2627). TMS = trimethylsilyl group.
